# DEC2 Serves as Potential Tumor Suppressor in Breast Carcinoma

**DOI:** 10.1155/2020/6053154

**Published:** 2020-10-10

**Authors:** Wentong Fang, Qian Li, Min Wang, Mingjie Zheng, Huirong Xu

**Affiliations:** ^1^Department of Pharmacy, First Affiliated Hospital of Nanjing Medical University, Nanjing 210029, China; ^2^Department of Breast Surgery, First Affiliated Hospital of Nanjing Medical University, Nanjing 210029, China; ^3^Department of Pathology, Zibo Central Hospital, Zibo 255036, China

## Abstract

**Background:**

Identification of new biomarkers can facilitate the development of effective therapeutic strategies in breast cancer (BC). Data from previous studies have revealed that differentiated embryonic chondrocyte gene (DEC) 1 and DEC2 might involve in the progression of various cancer types. We explored the expression profiles and function of DEC1/2 in BC patients in this study.

**Methods:**

The mRNA expression of DEC1/2 in BC patients and cell lines were taken from the Oncomine and Cancer Cell Line Encyclopedia database. The prognostic impacts of DEC1/2 were mined from the bc-GenExMiner and Kaplan–Meier plotter database. The impact of DEC1/2 genomic alterations on patient survival was calculated by cBioPortal. DEC2 protein expressions were confirmed by Western blotting (WB) in 10 pairs of BC samples. In addition, DEC2 sgRNA was constructed to confirm its affection on cell viability, invasion, and colony formation.

**Results:**

The DEC1 and DEC2 mRNA levels are both lower in BC tissues than normal tissues. DEC1/2 expression was high in progesterone receptor (PR) positive BC patients (*P* = 0.0023), but low in human epidermal growth factor receptor 2 (HER2) positive patients (*P* < 0.0001). Lower DEC2 mRNA level has significant association with more aggressive pathogenic grade (*P* < 0.0001) and worse overall survival (OS) of BC patients (*P* = 5.2 × 10^−6^). Subgroup analysis showed that low DEC2 level was correlated with worse OS in estrogen receptor (ER) positive BC (*P* = 0.008). DEC2 (*P* = 0.00029) alteration was significantly correlated with worse OS in BC patients. WB results also confirmed the lower DEC2 protein levels in BC samples than their paired normal tissues. And, DEC2 silencing by sgRNA resulted in a significant increasing in cell viability, invasion, and colony formation.

**Conclusion:**

DEC2 might serve as a tumor suppressor, and its disfunction may involve in the tumorigenesis and indicate bad clinical outcomes in BC patients.

## 1. Introduction

Breast cancer (BC) is the most common tumor among women globally. According to World Health Organization (WHO) Global Cancer Observatory (GLOBOCAN) [1, 2], BC has the highest age-standardized frequency (46.3 per 100,000) among all cancer types, which means 2.09 million new cases in 2018. Due to the development of gene detection and molecular subtype classification, endocrine therapy have formed for BC patients who expresses estrogen receptors (ER) and/or progesterone receptors (PR), while targeted therapy have used for patients with human epidermal growth factor receptor 2 (HER2) amplification [3]. These significantly improved the survival of some BC patients [4, 5]. Despite such progress in diagnosis and treatment, about 10% of all patients and about 70% of advanced patients died within 5 years after diagnosis [3]. Hence, it is necessary to identify new pathogenic genes in order to develop novel targeted therapy.

Differentiated embryonic chondrocyte gene (DEC) 1 and DEC2 are members of the basic helix-loop-helix (bHLH) transcription factor (TF) family and served as transcriptional repressors [6]. DEC1/2 can interact with TF2B, TBP, or TF2D or recruit a histone deacetylase at the E-box site [7, 8] and then regulate target genes transcription. DEC1/2 also interact with retinoid X receptor, myogenic TF, or C/EBP, in the E-box-independent manner [9].

Data from previous studies have revealed that DEC1/2 might be involved in the progression of many cancer types [2, 9–11]. DEC1 level was negatively related with tumor stage or differentiation grade in lung and esophageal cancer [12–14]. In colon, oral, liver, and brain cancers, DEC1 mRNA levels of tumor samples were higher than that of paired normal tissues [9–11]. The relation between DEC2 and cancer has also been explored. DEC2 expression is higher in human endometrial cancer (HEC) than in normal adjacent endometrial tissue [15]. All these indicated that DEC1/2 had some relation with the tumor initiation, progression, and outcome. However, their roles in BC have been rarely known up to now. In the current study, we used bioinformatical and molecular biological methods to explore the expression pattern and function of DEC1/2, in order to reveal their potential prognostic and therapeutic implications in BC.

## 2. Methods

### 2.1. Bioinformatics Analyses

The Oncomine cancer microarray online database (http://www.oncomine.org) [16] was used to check the DEC1/2 mRNA level in various cancers. When comparing the DEC1/2 mRNA level between tumor and normal samples, the statistic difference was defined as *P* values less than 0.01 and a fold change of more than 2 or less than -2.

The transcriptional expression of DEC1/2 in multifarious tumor cell lines was obtained from the Cancer Cell Line Encyclopedia (CCLE) database (http://portals.broadinstitute.org/ccle). The CCLE is an online encyclopedia of data collection, which provides public access to genomic data, analysis, and visualization for over 1100 cell lines.

Correlation analysis between the DEC1/2 mRNA level and clinicopathological parameters was done through Breast Cancer Gene-Expression Miner v4.4 (bc-GenEx- Miner v4.4) [17]. It is a statistical mining tool which can compare the DNA or RNA level of certain genes with clinical parameters and assess their predictive values in BC.

The Kaplan–Meier plotter online database (http://www.kmplot.com) was used to access the prognostic value of DEC1/2. It contains gene expression and survival information of 6234 BC patients [18, 19]. Patients are divided to two groups (high vs. low expression) depending on the DEC1/2 expression level, and the overall survival (OS) and relapse-free survival (RFS) between groups were compered by the Kaplan–Meier survival plot.

Genomic alteration contains gene mutations and copy number variance. The relation between DEC1/2 genomic alterations and clinical outcomes was analyzed by cBioPortal online database (http://www.cbioportal.org) [20, 21]. This database contains pathological and prognostic data of 9344 BC patients.

### 2.2. Cellular Biology Analysis

#### 2.2.1. Cell Culture

MCF-7 cells and T47D cells were, respectively, cultured in DMEM medium (Gibco, 11995-065) and RPMI 1640 medium (GIBCO 11875093) supplemented with 10% FBS (corning, 35015168), 100 mg/ml penicillin and 100 mg/ml streptomycin at 37°C in the presence of 5% CO_2_.

#### 2.2.2. Lentiviral Vectors and Lentivirus Production

DEC2 sgRNA plasmid and lentivirus are constructed as previously described [22]. Target sequences were as follows:

Control sgRNA: GCGAGGTATTCGGCTCCGCG

DEC2 sg1: GCTCGCCGCCGAGAACGACACGG

DEC2 sg2: ATCGCCCATTCAGTCCGACTTGG.

#### 2.2.3. MTS Assay

MCF-7 cells were seeded in triplicate in 96-well plates (1000 cells/well) in 200 *μ*l medium. After 48 h incubation, cells were replaced with 90 *μ*l fresh growth medium supplemented with 10 *μ*l MTS reagents (Abcam, ab197010), followed by incubation at 37°C for 1 h. OD absorbance values were measured at 490 nm using a 96-well plate reader (BioTek).

#### 2.2.4. Colony Formation Assay

MCF7 cells were seeded in a 6-well plate (2 × 103 cells/well) in 2 ml medium. The medium was changed every two days. After 7 days, the cells were fixed with 4% formaldehyde for 10 minutes at room temperature, stained for 10 minutes with 0.5% crystal violet, and then washed several times with distilled water. Once dried, the plates were scanned.

#### 2.2.5. Cell Invasion Assay

BD BioCoat Matrigel Invasion Chambers (354480) were used for MCF7 and T47D cell invasion assays according to manufacturer's instructions. MCF7 and T47D cell suspensions were seeded at 4 × 104 cells in each chamber in triplicate and incubated for 12 h in an incubator at 37°C in 5% CO_2_. Cells on the lower surface of the membrane were stained with crystal violet and counted under an EVOS XL Core microscope (AMEX1000, Thermo Fisher Scientific).

#### 2.2.6. Western Blot Analysis and Antibodies

EBC buffer (50 mM Tris pH 8.0, 120 mM NaCl, 0.5% NP40, 0.1 mM EDTA, and 10% glycerol) supplemented with complete protease inhibitor (Roche Applied Biosciences) was used to harvest whole cell lysates at 4°C. Cell lysates concentration was measured by Protein Assay Dye (Bio-Rad). Equal amount of cell lysates was resolved by SDS-PAGE. Rabbit DEC2 antibody (ab82825) was from Abcam, Mouse *α*-tubulin antibody (Cell Signaling, 3873). Peroxidase conjugated goat anti-mouse secondary antibody (31430) and peroxidase conjugated goat anti-rabbit secondary antibody (31460) were from Thermo Scientific.

#### 2.2.7. Statistical Analysis

To assess the statistical significance of a difference between two conditions, we used unpaired two-tailed student's *t*-test. All graphs depict mean ± SEMunless otherwise indicated. Statistical significances are denoted as n.s. (not significant; *P* > 0.05), ∗*P* < 0.05, ∗∗*P* < 0.01, ∗∗∗*P* < 0.001.

## 3. Results

### 3.1. Transcriptional Levels of DEC1 and DEC2 in BC

**Figure 1:**
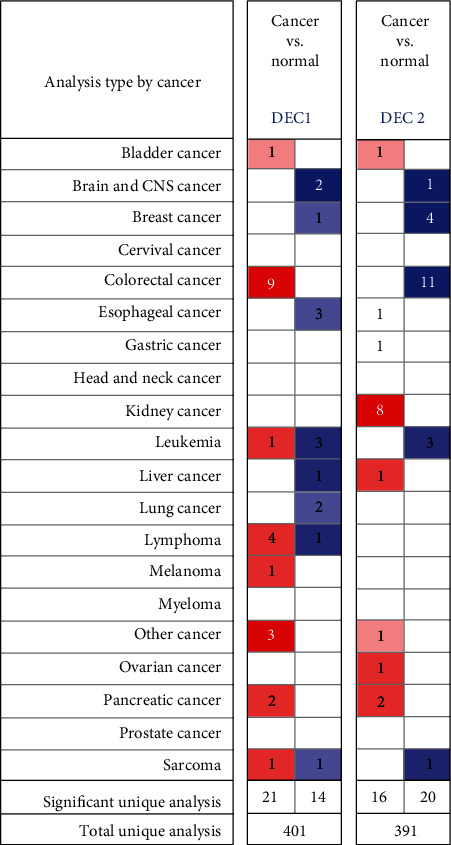
Transcriptional levels of DEC1/2 in different cancer types. Notes: This graphic was obtained from Oncomine (http://www.oncomine.org) which indicates the numbers of datasets with significant overexpression (red) or downexpression (blue) of SHARPs at transcriptional levels in cancer tissues compared with those in corresponding normal tissues. Cell color was determined by the best gene rank percentile for the analyses within the cell, and the gene rank was analyzed by the percentile of target genes at the top of all genes measured in each research. CNS: central nervous system.

We used the Oncomine database to examine the mRNA expression differences of DEC1/2 between tumor and normal samples in different cancer types. There are 401 datasets that contain DEC1 mRNA expression information. Twenty-one analyses showed higher DEC1 expression in tumors than normal samples, while 14 researches showed lower DEC1 expression in tumors than normal tissues. Regarding BC, only one study revealed lower expression of DEC1 in cancer tissues than normal tissues (Figure 1). But there are 7 subgroups which compared the DEC1 mRNA level between BC and normal tissues. All these 7 subgroups had significant *P* values, but only 1 dataset reached the defined criteria for the fold changes. In the Ma 4 research, DEC1 expression in ductal breast carcinoma in situ stroma was 2.008-fold lower than normal breast tissues. Unfortunately, there were only 25 samples included in this study (Table 1).

There are 391 datasets that contain DEC2 mRNA expression information. Compared to normal samples, DEC2 mRNA levels were upregulated in tumors as demonstrated in 16 studies and downregulated in 20 analyses. Regarding BC, four studies revealed lower expression of DEC2 in tumors than normal tissues (Figure 1). And, 2 datasets had significant *P* values, but no study reached the defined criteria for the fold changes (Table 1).

**Figure 2:**
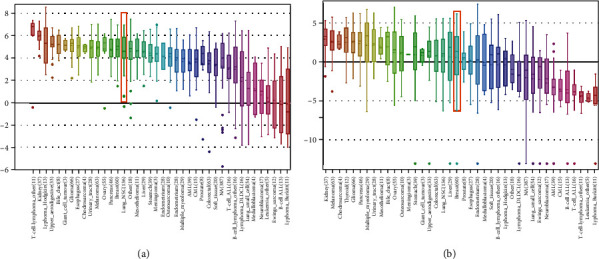
DEC1/2 were distinctively expressed in breast cancer cell lines from the Cancer Cell Line Encyclopedia analysis. Notes: The mRNA expression levels of DEC1 (a) and DEC2 (b) in breast cancer cells, ranks in the 20th and 15th among all cancer cell types (shown in red frame). CML: chronic myelocytic leukemia; NSC: non-small cell; DLBCL: diffuse large B cell lymphoma; AML: acute myelocytic leukemia; NA: not applicable.

We also explored the mRNA levels of DEC1 and DEC2 in various human tumor cell lines by mining the CCLE database. The DEC1/2 mRNA expression have been reported in 40 different cancer types and more than 1000 human cell lines. We calculated the average mRNA level of DEC1 in 60 different breast cancer cell lines, and it listed in the 15th position among all cancer types. The average mRNA level of DEC2 listed in the 20th position among 40 cancer types (Figure 2).

### 3.2. Correlation between DEC1/2 Transcriptional Level and Molecular Subtype in BC

**Figure 3:**
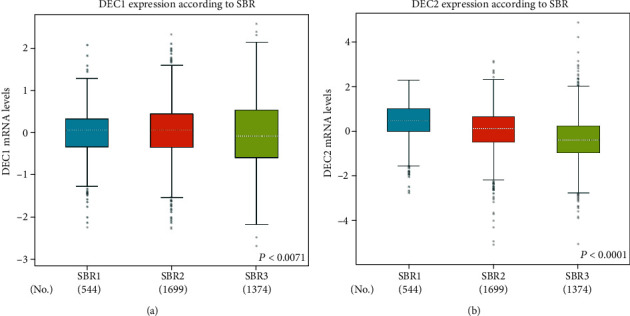
Correlation of mRNA expression of DEC1/2 with SBR grade status. Notes: Global significant differences of DEC1 (a) and DEC2 (b) between groups were assessed by Welch's test to generate *P* values, along with the Dunnett–Tukey–Kramer's tests for pairwise comparison when a global significant difference exists. SBR: Scarff, Bloom, and Richardson.

The relation between DEC1/2 expression level and molecular subtype in BC was mined from the bc-GenExMiner database. In Table 2, the DEC1/2 mRNA level has no relation with nodal status and ER expression. Patients >51 years have lower DEC2 mRNA levels (*P* < 0.0001), but higher DEC1 mRNA levels (*P* = 0.0208) than patients aged ≤51 years. DEC1/2 expression was higher in PR positive BC patients (*P* = 0.0023), but lower in HER2 positive patients (*P* < 0.0001). In triple-negative breast cancer (TNBC) patients, DEC2 mRNA levels were significantly decreased (*P* < 0.0001), while DEC1 mRNA levels were significantly increased (*P* = 0.0196). Furthermore, lower DEC2 mRNA level was significant association with more aggressive pathological grade (Scarff, Bloom, and Richardson grade) (*P* < 0.0001, Figure 3).

### 3.3. The Relation between DEC1/2 mRNA Level and Clinical Outcomes

**Figure 4:**
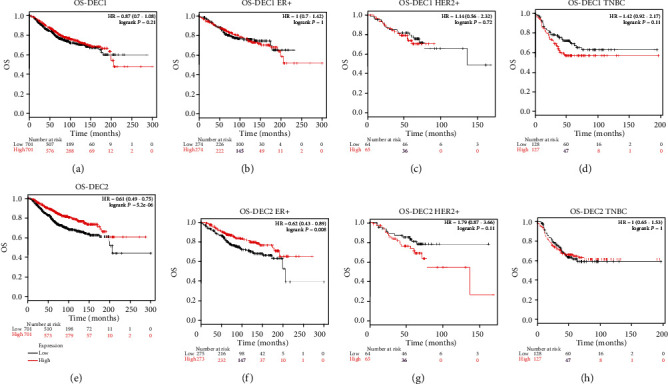
Prognostic value of mRNA levels of DEC1/2 in breast cancer patients (OS in the Kaplan–Meier plot). Notes: The impact of DEC1 (a) and DEC2 (e) on OS of breast cancer patients. The impact of DEC1 (b) and DEC2 (f) on OS in ER-positive subtype. The impact of DEC1 (c) and DEC2 (g) on OS in HER positive subtype. The impact of DEC1 (d) and DEC2 (h) on OS in triple negative subtype. OS: overall survival; RFS: relapse-free survival.

The relation between DEC1/2 mRNA level and clinical outcomes was determined by the Kaplan–Meier plotter survival analysis. DEC2 mRNA level was positively correlated with overall survival (OS) of BC patients (*P* = 5.2 × 10^−6^, Figure 4(a)). Subgroup analysis showed that high DEC2 expression indicated good OS in ER positive BC (*P* = 0.008, Figure 4(b)). There was no significantly correlation between DEC1 mRNA level and OS of BC.

### 3.4. DEC1/2 Gene Alteration Affect BC Patient Survival

**Figure 5:**
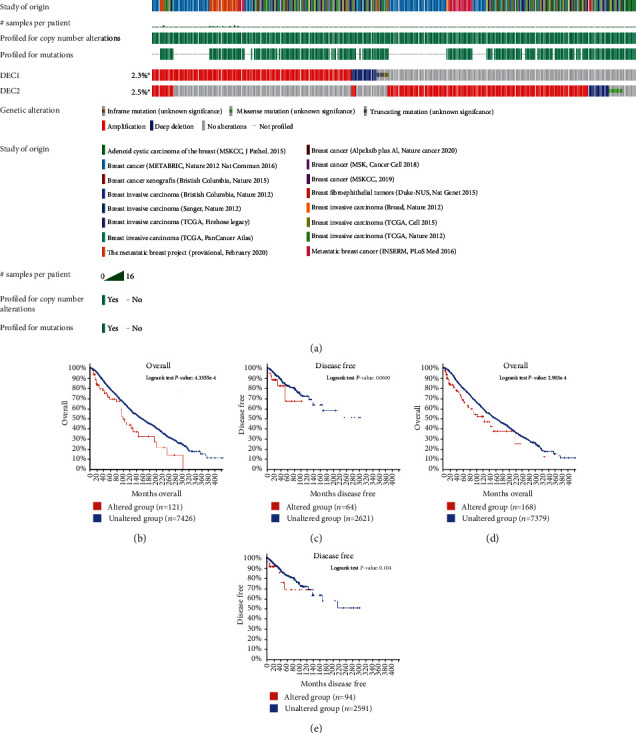
Genetic alterations of DEC1/2 gene expression and their association with patient survival in breast invasive carcinoma (cBioPortal). Notes: (a) Oncoprint in cBioPortal represented the proportion and distribution of samples with alterations in DEC1 and DEC2. The figure was cropped on the right to exclude samples without alterations. The Kaplan–Meier plots comparing OS in cases with/without DEC1 (b) and DEC2 alterations (d). The Kaplan–Meier plots comparing DFS in cases with/without DEC1 (c) and DEC2 (e) alterations. DFS: disease-free survival; OS: overall survival; TCGA: The Cancer Genome Atlas.

The incidence of DEC1 and DEC2 gene alteration is 2.3% and 2.5% in BC patients separately (Figure 5(a)). The effect of gene mutation on clinical outcome was analyzed by the Kaplan–Meier plot and log-rank test. DEC1 (*P* = 0.044, Figure 5(b)) and DEC2 (*P* = 0.00023, Figure 5(d)) alteration was significantly correlated with worse OS of BC patients, while these alterations have no relation with RFS (Figures 5(c) and 5(e)).

### 3.5. DEC2 Serves as a Potential Tumor Suppressor in BC

**Figure 6:**
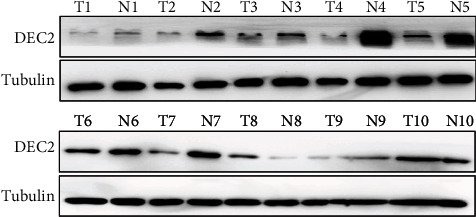
The expression of DEC2 in breast cancer samples. Notes: DEC2 protein levels were determined by the Western blot analysis in primary breast cancer and paired normal tissues.

**Figure 7:**
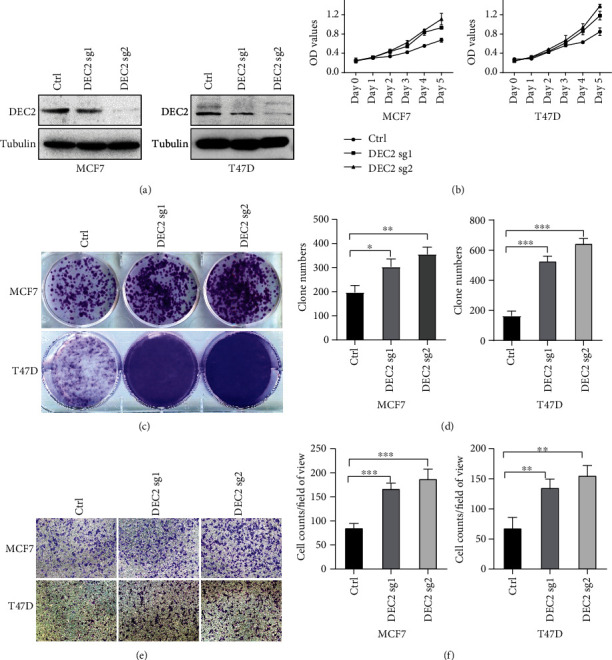
The function of DEC2 in breast cancer cells. Notes: (a) Immunoblot of cell lysates of MCF7 and T47D cells infected with lentivirus encoding either DEC2 sgRNA (1, 2) or control (Ctrl). (b) Quantification of cell proliferation assays of MCF7 and T47D cells infected with lentivirus encoding either DEC2 sgRNA (1, 2) or control (Ctrl). (c) Colony formation assay of MCF7 and T47D cells infected with lentivirus encoding either DEC2 sgRNA (1, 2) or control (Ctrl). (d) Quantification of clones of MCF7 and T47D cells infected with lentivirus encoding either DEC2 sgRNA (1, 2) or control (Ctrl). (e) Cell invasion assays of MCF7 and T47D cells infected with lentivirus encoding either DEC2 sgRNA (1, 2) or control (Ctrl). (f) Quantification of cell invasion assays of MCF7 and T47D cells infected with lentivirus encoding either DEC2 sgRNA (1, 2) or control (Ctrl).

Then, we examined the protein level of DEC2 in 10 pairs of human BC samples with their matching adjacent noncancerous tissues by Western blotting. Seven of these ten tumor samples had significantly lower DEC2 protein levels than their paired normal tissues (Figure 6). We further determined whether low DEC2 expression means high malignancy and bad prognosis. We performed these experiments in ER-positive MCF7 and T47D cells. Firstly, we silenced DEC2 by sgRNAs.  Figure 7(a) showed silencing efficiency in MCF7 and T47D cells upon DEC2 knockdown by DEC2 sg1 and sg2 as compared with scramble (sgCtrl). DEC2 silencing resulted in a significant increasing in cell viability (MTS assays) (Figure 7(b)), colony formation (Figures 7(c) and 7(d)) and cell invasion (Figures 7(e) and 7(f)).

## 4. Discussion

Benefit from the rapid development of gene detection and the Hadoop analysis, dozens of genes have been identified to take part in the initiation, progression, and outcome of BC. These advances finally converted to the improvement in the therapy and outcome [23]. Yet for all that, the exact pathogenesis of BC has not been clearly explicated. Hence, it is necessary to further mine candidate genes which have diagnostic and therapeutic implications. In this study, we revealed the expression pattern and function of DEC1/2 in BC by bioinformatical and molecular biological assays. Our results indicate that DEC2 might serve as a tumor suppressor, and its disfunction may involve in the tumorigenesis and indicate bad clinical outcomes in BC patients.

The relationship between DEC1 and cancer has been widely explored, but the results are contradictory. Previous studies have showed that DEC1 expression is higher in colon, oral, liver, and brain tumors tissues than in adjacent normal tissues [9–11] but inversely correlated with the tumor stage or differentiation grade in oral, lung, and esophageal cancer [12–14]. Previous in vitro studies have explored the role of DEC1 in BC, but no consensus has been reached. In the study of Sethuraman et al. [24], DEC1 has suggested as a prometastasis factor, and promotes tumor cell survival and migration by modulating exosomic secretion of heparin-binding epidermal growth factor (HBEGF) [24]. But in the study of Asanoma et al., DEC1 overexpression has been shown to inhibit cell proliferation, migration, or invasion and to induce cellular senescence [9]. In our research, DEC1 expression is lower in cancer tissues than in normal tissues according to the Oncomine cancer microarray online database (Figure 1), and DEC1 mutation significantly affects OS of BC patients (Figure 5(b)). But, further studies showed DEC1 mRNA levels have no relation with grade SBR (Figure 3(a)) and OS (Figure 4(a)). Therefore, we think DEC1 might not be a robust prognostic factor in BC.

Little studies have explored the function of DEC2 in cancer. In the study of Yunokawa et al., DEC2 was significantly higher in HEC compared with those in normal endometria [15].

Some researches have suggested that DEC2 expression was positively related to tumor progression, but some other studies showed negative correlation [9]. In TNBC, DEC2 is reported to suppress metastasis by direct promoting the degradation of HIF1a and HIF2a and directly suppressing *CCND1* transcription [5]. These observations are consistent with our findings. In our research, cell viability, invasion, and colony formation of MCF7 and T47D cells significantly increased when DEC2 was knocked down (Figure 7). In in silico data-mining approaches, DEC2 levels are lower in cancer than normal tissues (Figure 1), which was also confirmed by our Western blotting analysis (Figure 6). These showed potential significance of DEC2 in BC.

A previous study about TNBC has showed stronger expression of DEC2 that correlated with better prognoses, including metastasis-free survival [5], which is coincident with our research. In our in silico analysis, higher DEC2 mRNA expression was associated with lower SBR (Figure 3(b)) and better OS (Figure 4(e)) in BC patients. Mutation in DEC2 led to poor OS (Figure 5(c)). Further, our analyses revealed that the high mRNA level of DEC2 was correlated with a favorable OS in patients with ER-positive BC subtype. We inferred that the reason, at least in part, might be due to ER positive BC is less invasive and malignant than ER negative BC. All these results confirmed that DEC2 might serve as a tumor suppressor, and its disfunction may involve in the tumorigenesis and indicate bad clinical outcomes in BC patients.

There are some limitations in our research. On the one hand, we only explored the relation between DEC1/2 mRNA level and patient survival. We cannot find the DEC1/2 protein expression profile through in silico analysis. Hence, we tested the DEC2 protein level in 10 pairs of human samples. But, the DEC1/2 protein level needs to be confirmed in a large scale of human BC samples. On the other hand, the exact molecular mechanism of DEC2 has not been revealed. More physiological and molecular validation is needed to confirm the function of DEC2 in cancer initiation and development.

## 5. Conclusion

In the current study, we revealed the expression pattern and function of DEC1/2 by using bioinformatical and molecular biological methods. Our findings contribute to the systematic understanding of the biological functions of DEC1/2 in BC as well as provide the evidence that DEC2 might serve as a tumor suppressor, and its disfunction may involve in the tumorigenesis and indicate bad clinical outcomes in BC patients.

## Figures and Tables

**Table 1:** Datasets of DEC1/2 in breast cancer (Oncomine database).

Gene	Dataset	Normal (cases)	Tumor (cases)	Fold change	*t*-test	*P* value
DEC1	Ma 4	Breast (14)	Ductal breast carcinoma in situ (9)	1.36	4.35	0.000161
		Breast (14)	Ductal breast carcinoma in situ stroma (11)	2.008	3.125	0.002
		Breast (14)	Invasive ductal breast carcinoma stroma (9)	1.889	2.347	0.015
		Breast (14)	Invasive ductal breast carcinoma epithelia (9)	1.568	2.707	0.011
	TCGA breast	Breast (61)	Invasive lobular breast carcinoma (36)	1.401	3.405	0.000516
		Breast (61)	Invasive breast carcinoma (76)	1.205	2.058	0.021
		Breast (61)	Invasive ductal breast carcinoma (389)	1.196	2.481	0.007

DEC2	Finak breast	Breast (6)	Invasive breast carcinoma (53)	1.37	1.88	0.044
	Karnoub breast	Breast (15)	Invasive ductal breast carcinoma (7)	1.826	2.463	0.012

**Table 2:** Datasets of DEC1/2 in breast cancer from bc-GenExMiner v4.1.

Variables		DEC1			DEC2		
		*n*	mRNA	*P* value	*n*	mRNA	*P* value
Age (years)	≤51	1099	—	<0.0001	1099	—	0.0208
	>51	3208	↑		3208	↓	

Nodal status	Negative	2415	—	0.1487	2415	—	0.9097
	Positive	1645	—		1646	—	

ER (IHC)	Negative	551	—	0.0525	551	—	0.0547
	Positive	3911	—		3911	—	

PR (IHC)	Negative	828	—	<0.0001	828	—	0.0023
	Positive	3498	↑		3498	↑	

HER2 (IHC)	Negative	3582	—	0.0002	3582	—	<0.0001
	Positive	661	↓		661	↓	

Triple-negative status (IHC)	Not	4119		<0.0001	4119	—	0.0196
	TNBC	317	↑		317	↓	

Basal-like status	Not	3836	—	<0.0001	3836	—	0.9056
	Basal-like	832	↓		832	—	

## Data Availability

The data used to support the findings of this study are available from the corresponding author upon request.
